# Association between the skeletal muscle mass to visceral fat area ratio and metabolic dysfunction‐associated fatty liver disease: A cross‐sectional study of NHANES 2017–2018

**DOI:** 10.1111/1753-0407.13569

**Published:** 2024-05-16

**Authors:** Zhiliang Mai, Yinfei Chen, Hua Mao, Lisheng Wang

**Affiliations:** ^1^ Department of Gastroenterology Zhujiang Hospital, Southern Medical University Guangzhou China; ^2^ Department of Gastroenterology Shenzhen People's Hospital (The Second Clinical Medical College, Jinan University; The First Affiliated Hospital, Southern University of Science and Technology) Shenzhen China; ^3^ Department of Endocrinology Zhujiang Hospital, Southern Medical University Guangzhou China

**Keywords:** MASLD, NHANES, skeletal muscle mass to visceral fat area ratio

## Abstract

**Background and Aims:**

Previous studies have shown that sarcopenic obesity (SO) was associated with nonalcoholic fatty liver disease (NAFLD). However, research is limited in the context of the NAFLD renamed as metabolic dysfunction‐associated steatotic liver disease (MASLD) defined by updated diagnostic criteria. The aim of this study was to use the index skeletal muscle mass to visceral fat area ratio (SVR) to describe SO in a large and representative US population (National Health and Nutrition Examination Survey 2017–2018) of adults and investigate their association with MASLD.

**Methods:**

A total of 2087 individuals were included in the analysis. SVR was calculated according to the measurement of dual‐energy x‐ray absorptiometry and MASLD was diagnosed with controlled attenuation parameter scores and cardiometabolic risk factors. SVR was divided into tertiles. Logistic regression adjusted for confounders was used to evaluate the association between SVR and MASLD. Several sensitivity analyses were performed to test the robustness of our findings.

**Results:**

In a multivariate logistic regression analysis, a significant association between SVR and MASLD was shown (odds ratio [OR]: 3.11, 95% confidence interval [CI]: 1.31–7.39, *p* = .010 for middle levels of SVR; OR: 3.82, 95% CI: 1.45–10.08, *p* = .007 for lowest levels of SVR). The sensitivity analyses confirmed that the association was robust.

**Conclusion:**

Our findings imply that decreased SVR is linked to MASLD.

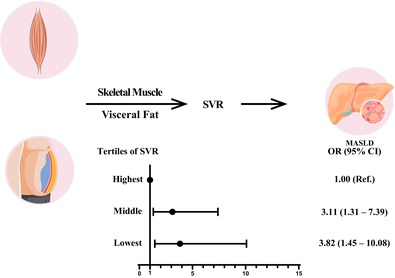

## Graphical abstract

Highlights

- Using data from the National Health and Nutrition Examination Survey, our study is among the first to investigate the relationship between sarcopenic obesity, represented by the skeletal muscle mass to visceral fat area ratio (SVR), and metabolic dysfunction‐associated steatotic liver disease (MASLD) in the US population.
- Measurements for SVR were obtained using dual‐energy x‐ray absorptiometry, and MASLD was diagnosed with controlled attenuation parameter scores and cardiometabolic risk factors.
- The present study revealed that lower levels of SVR were strongly associated with an increased risk of MASLD.

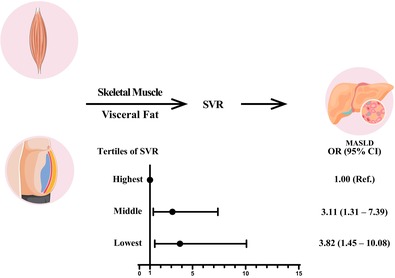

## 1 INTRODUCTION

Metabolic dysfunction‐associated steatotic liver disease (MASLD), previously known as nonalcoholic fatty liver disease (NAFLD) or metabolic dysfunction‐associated fatty liver disease (MAFLD),^1^ represents an important global public health problem. With NAFLD affecting approximately 25%–30% of the world's adult population,^2^ it is regarded as part of a multisystem disease that is associated with higher risk of liver‐specific complications and various extrahepatic diseases.^3^, ^4^, ^5^, ^6^ Moreover, it is closely associated with other chronic diseases that have rapidly increased in the last years, such as diabetes,^7^ obesity,^8^ and hypertension.^9^ Consequently, its prevalence is expected to rapidly rise as well.^10^, ^11^ Therefore, further research is needed to identify emerging risk factors contributing to the disease, which might offer better insight into how best to identify high‐risk individuals and develop prevention strategies.

Sarcopenia, defined as the loss of skeletal muscle mass, has been found to be a risk factor for NAFLD and associated with its worse clinical outcome.^12^, ^13^ The link to sarcopenia is plausible, considering the function of skeletal muscle in energy homeostasis and insulin‐mediated glucose metabolism.^14^ Similarly, obesity, especially visceral obesity, plays an important role in NAFLD.^15^, ^16^, ^17^ When sarcopenia coexists with obesity, this combination leads to a multifactorial condition called sarcopenic obesity (SO), which is characterized by decreased muscle mass and increased fat mass.^18^ This interaction may form a vicious cycle, as adipose tissue can cause damage to muscle homeostasis, thereby leading to muscle atrophy and a reduced regeneration capacity.^19^, ^20^ Moreover, loss of muscle mass can result in a lack of exercise, further increasing fat mass. Therefore, sarcopenia and obesity may have a synergistic effect on NAFLD. Nevertheless, the interactions between NAFLD, sarcopenia, and obesity remain insufficiently explored.

Considering the interaction of sarcopenia with obesity, along with the stronger association of visceral fat area (VFA) with metabolic syndrome than body mass index (BMI) and waist circumference,^21^ several studies have demonstrated that the index skeletal muscle mass to visceral fat area ratio (SVR) is a promising index to describe SO and evaluate the risks of diabetes^22^ and cardiovascular diseases.^23^ A previous study has also illustrated a higher risk of incident NAFLD in individuals with low SVR.^24^ However, the study was limited as it merely used bioelectrical impedance analyzer (BIA) for body composition measurements and liver ultrasound for the diagnosis of NAFLD, limiting the accuracy of the association between SVR index and NAFLD. Also, the association between SVR index and MASLD remains unclear after the new nomenclature for NAFLD.

Hence, to address these limitations, this study aimed to use more accurate methods to examine the associations between SVR and MASLD.

## 2 METHODS

### 2.1 Study population

The National Health and Nutrition Examination Survey (NHANES) is a representative sample of US civilian, noninstitutionalized population and is selected with a complex multistage probability design. In the NHANES 2017–2018 cycle, vibration‐controlled transient elastography was introduced for the first time to estimate hepatic steatosis by using the controlled attenuation parameter (CAP). Participants aged 12 years and older were eligible for elastography measurements, with exceptions including those who (a) were unable to lie down on the exam table; (b) were pregnant or unsure if pregnant; (c) had an implanted electronic medical device; and (d) were wearing a bandage or had lesions on the right side of their abdomen by the ribs. CAP has been extensively evaluated for its accuracy to assess liver steatosis. Consequently, the present study focused exclusively on the NHANES 2017–2018 circle. The exclusion criteria of the present study were as follows: (a) participants younger than 18 years or older than 60 years because the individuals under 60 years were eligible for dual‐energy x‐ray absorptiometry (DXA); (b) participants whose elastography examination status was not performed, ineligible or partial; (c) insufficient data for assessment of appendicular skeletal muscle mass index (ASMI) and VFA; (d) participants who had positive serum hepatitis B surface antigen or positive serum hepatitis C antibody; and (e) excessive alcohol users. The National Center for Health Statistics created public use files, and NHANES for the 2017–2018 cycle was approved by the National Center for Health Statistics Research Ethics Review Board, which waived the need for informed consent for the use of publicly available data.

### 2.2 Definition of MASLD

MASLD was diagnosed by meeting the criterion of a median CAP score of ≥285 dB/m, which has an optimized sensitivity of 80% and specificity of 77% for detecting hepatic steatosis^25^ in the absence of any other possible causes of chronic liver disease. Diagnosis also required the presence of at least one of following cardiometabolic risk factors including overweight/obesity/central obesity, hyperglycemia or diabetes, high blood pressure, high triglycerides, and reduced high‐density lipoprotein cholesterol (HDL‐C): (a) BMI ≥25 kg/m^2^ or waist circumference (WC) >94 cm (male) 80 cm (female); (b) fasting serum glucose ≥5.6 mmol/L [100 mg/dL] or glycated hemoglobin ≥5.7% [39 mmol/L] or diabetes or treatment for diabetes; (c) blood pressure ≥130/85 mm Hg or specific antihypertensive drug treatment, (d) plasma triglycerides ≥1.70 mmol/L [150 mg/dL] or lipid lowering treatment, and (e) plasma HDL‐C ≤1.0 mmol/L [40 mg/dL] (male) and ≤1.3 mmol/L [50 mg/dL] (female) or lipid‐lowering treatment.^1^

### 2.3 DXA measurement and definition of SVR

The DXA scans provided soft tissue measurements for the total body, as well as both arms and legs. Using these measurements, the ASMI was calculated as the sum of lean mass for all four extremities (arms and legs, kg). The SVR was defined by the following formula: appendicular lean mass divided by visceral fat area (kg/cm^2^). SVR was then divided into tertiles as highest tertile, middle tertile, and lowest tertile.

### 2.4 Covariate

Information on demographic characteristics was collected through self‐reports. Ethnicity was self‐reported and categorized as: Non‐Hispanic White, Non‐Hispanic Black, Hispanic, and other races. Furthermore, an individual's sedentary behavior was defined if they answered “no” to vigorous or moderate physical activity in the previous month. Self‐reported smoking status was classified as never, former, or current. Those who reported “smoking now and smoked at least 100 cigarettes” were categorized as current smokers, whereas participants who had quit smoking were categorized as former smokers. To assess weekly alcohol intake, we classified alcohol consumption as follows: (a) nondrinker defined as who answered “no” to “Ever had a drink of any kind of alcohol?”; (b) light alcohol user: less than 140 g/week for females and less than 210 g/week for males; (c) moderate alcohol user: 140–350 g/week for females and 210–420 g/week for males; and (d) excessive alcohol user: more than 350 g/week for females and more than 420 g/week for males.^26^ The amount of daily energy intake, daily carbohydrate intake, daily fat intake, and daily protein intake was captured by two 24‐h dietary recalls.

### 2.5 Statistical analysis

Examination sample weights, accounting for nonresponse, noncoverage, and unequal selection probabilities for certain categories of the population, were incorporated to produce national estimates for all analyses. Differences in demographic and clinical characteristics between comparison groups were determined by one‐way analysis of variance for continuous variables and the chi‐square test for categoric variables. Post hoc multiple comparison analysis with Bonferroni correction was performed for significant results.

Associations between SVR and MASLD were first investigated using multivariable logistic regression models by tertiles of SVR. Individuals in the highest tertile were used as the reference group. The results are reported as odds ratios (OR) and their 95% confidence intervals (CI). Model 1 adjusted for age and sex. Model 2 added education, race, marital status, poverty‐income ratio, BMI, WC, smoking status, and alcohol consumption. Model 3 added sedentary behavior, total energy intake per day, total carbohydrate intake per day, total fat intake per day, total protein intake per day, total cholesterol (TC), and low‐density lipoprotein cholesterol (LDL‐C). Missing data for categorical variables were classified as unknown in our models.

Besides, to further explore the dose–response association between SVR and risk of MASLD, we used restricted cubic splines. Knots were placed at the 10th, 50th, and 90th percentiles of the exposure distribution. Nonlinearity in the relationship was assessed by a Wald test. If the nonlinearity was confirmed, we then estimated the inflection point, defined as the exposure value at which the maximum significant risk reduction was observed.^27^

### 2.6 Sensitivity analyses

Several sensitivity analyses were performed. First, we redefined MASLD using a median CAP of ≥263 dB/m for detecting the presence of 5% steatosis.^25^ Second, considering that body compositions differ according to sex, age and BMI, we stratified participants according to sex, age (<35 years and ≥35 years), and BMI (<30 kg/m^2^ and ≥30 kg/m^2^). Additionally, we performed tests of interaction regarding sex, age, and BMI to examine whether the association between SVR and MASLD varied across these subgroups. Third, missing values in BMI, WC, total energy intake per day, total carbohydrate intake per day, total fat intake per day, total protein intake per day, TC, and LDL‐C were imputed with multiple imputation, followed by a reanalysis. Fourth, hepatic steatosis was categorized into four grades based on CAP as follows^28^: (a) S0 (CAP ≤269 dB/m); (b) S1 (269 < CAP ≤ 288 dB/m); (c) S2 (288 < CAP ≤313 dB/m); and (d) S3 (CAP <313 dB/m). Subsequently, the prevalence of different grades of hepatic steatosis by SVR tertiles was calculated. Finally, Spearman's rank correlation coefficients between CAP and SVR was calculated.

Two‐sided *p* < .05 was considered statistically significant. All analyses were conducted using SAS version 9.4 (SAS Institute) and R statistical software version 4.0 (R Project for Statistical Computing).

## 3 RESULTS

### 3.1 Clinical and demographic characteristics of the study population

**FIGURE 1:**
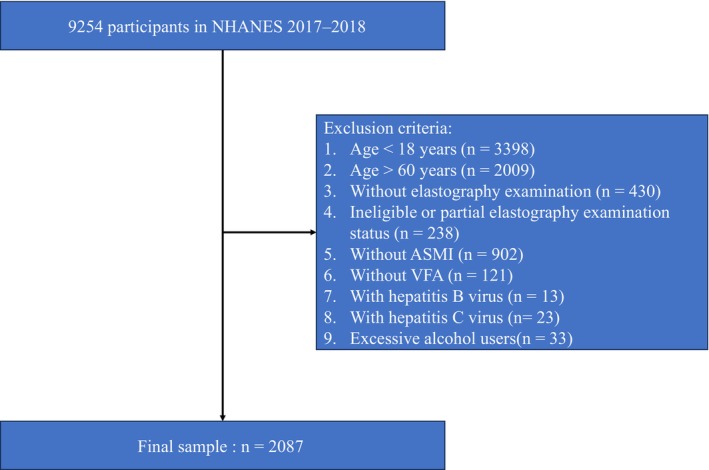
Flow chart of the participants' selection from NHANES 2017–2018. ASMI, appendicular skeletal muscle mass index; NHANES, National Health, and Nutrition Examination Survey; VFA, visceral fat area.

There were 9254 participants from 2017 to 2018 included in this study. After removing 7167 participants due to the exclusion criteria, 2087 participants were finally enrolled (Figure 1). The survey‐weighted characteristics of participants by tertiles of SVR are presented in Table 1. The mean (SE) age of the study participants was 37.34 (0.57) years, and 50.59% were males. Of 2087 participants, 633 were identified suspected MASLD cases (sample‐weighted prevalence = 27.4%).

**TABLE 1:** Characteristics of the population by tertiles of SVR.

	Highest (*n* = 700)	Middle (*n* = 693)	Lowest (*n* = 694)	Total (*n* = 2087)	Overall *p*	Pairwise		
						Highest vs. Middle	Highest vs. Lowest	Middle vs. Lowest
Age (years)	31.30 (0.98)	37.22 (0.89)	44.35 (0.82)	37.34 (0.57)	*p* < .001	*p* = .001	*p* < .001	*p* = .001
Gender (%)					*p* < .001	*p* = .770	*p* = .001	*p* = .001
Male	59.23 (3.19)	57.98 (3.20)	33.82 (5.16)	50.59 (2.37)				
Female	40.77 (3.19)	42.02 (3.20)	66.18 (5.16)	49.41 (2.37)				
Race (%)					*p* < .001	*p* = .001	*p* < .001	*p* = .189
Non‐Hispanic White	62.75 (4.79)	50.23 (4.88)	57.18 (3.83)	57.14 (3.28)				
Non‐Hispanic Black	17.30 (3.40)	8.97 (1.84)	5.07 (0.91)	10.79 (1.79)				
Hispanic	12.42 (3.11)	25.67 (5.57)	21.83 (3.22)	19.50 (3.57)				
Other races	7.53 (1.31)	15.13 (3.06)	15.92 (3.19)	12.57 (2.00)				
BMI, kg/m^2^	24.21 (0.32)	28.52 (0.39)	31.31 (0.51)	27.83 (0.27)	*p* < .001	*p* < .001	*p* < .001	*p* = .003
WC, cm	83.87 (0.83)	96.72 (0.87)	104.69 (1.17)	94.57 (0.70)	*p* < .001	*p* < .001	*p* < .001	*p* < .001
Marital status					*p* = .001	*p* = .004	*p* = .002	*p* = .148
Single	53.50 (4.30)	42.38 (4.13)	42.33 (6.18)	46.49 (2.98)				
Married	35.20 (4.31)	53.75 (4.24)	57.06 (6.11)	47.94 (2.97)				
Unknown	11.30 (2.31)	3.87 (0.99)	0.62 (0.39)	5.57 (0.96)				
Educational level (%)					*p* < .001	*p* = .001	*p* < .001	*p* = .030
Less than high school	0.41 (0.32)	3.38 (1.16)	6.03 (1.20)	3.14 (0.72)				
High school	3.64 (0.96)	9.47 (2.11)	7.74 (1.96)	6.74 (0.83)				
More than high school	84.65 (2.32)	83.28 (3.23)	85.62 (1.78)	84.55 (1.35)				
Unknown	11.30 (2.31)	3.87 (0.99)	0.62 (0.39)	5.57 (0.96)				
Poverty‐income ratio (%)					*p* = .556			
<1.3	16.81 (2.68)	23.74 (2.75)	21.70 (2.93)	20.51 (1.67)				
1.3–1.8	8.00 (2.24)	8.12 (1.62)	9.36 (1.93)	8.48 (1.38)				
>1.8	66.28 (3.17)	59.64 (4.84)	58.05 (3.70)	61.58 (2.10)				
Unknown	8.91 (1.83)	8.50 (2.17)	10.90 (3.21)	9.43 (1.42)				
Sedentary behavior (%)					*p* = .725			
No	56.77 (3.63)	56.39 (3.41)	52.77 (4.57)	55.36 (2.43)				
Yes	43.04 (3.75)	43.61 (3.41)	47.15 (4.57)	44.55 (2.45)				
Unknown	0.18 (0.19)	0 (0)	0.08 (0.08)	0.09 (0.07)				
TC, mg/dL	174.97 (2.99)	191.09 (2.76)	191.87 (2.68)	185.34 (1.67)	*p* = .001	*p* = .002	*p* = .003	*p* = 1.000
LDL‐C, mg/dL	99.97 (2.59)	116.75 (2.48)	116.56 (2.20)	110.36 (1.47)	*p* < .001	*p* < .001	*p* = .001	*p* = 1.000
Total energy intake per day, kcal/day	2176.35 (70.57)	2124.49 (70.82)	2017.10 (62.8)	2107.08 (39.32)	*p* = .288			
Total carbohydrate intake per day, g/day	250.77 (9.31)	238.25 (9.45)	237.91 (9.11)	242.78 (5.35)	*p* = .377			
Total protein intake per day, g/day	86.36 (3.81)	84.27 (3.06)	76.30 (2.50)	82.33 (1.88)	*p* = .077			
Total fat intake per day, g/day	86.07 (3.05)	85.18 (3.08)	80.09 (2.89)	83.77 (1.74)	*p* = .351			
Cardiometabolic risk factors (yes), %								
Overweight/obesity/central obesity	47.39 (5.61)	84.14 (2.73)	93.53 (2.51)	73.63 (2.93)	*p* < .001	*p* < .001	*p* < .001	*p* = .067
Hyperglycemia or diabetes	31.59 (3.52)	57.11 (3.77)	73.04 (3.16)	52.82 (2.60)	*p* < .001	*p* < .001	*p* < .001	*p* = .004
High blood pressure	14.58 (3.74)	33.06 (3.37)	40.79 (4.41)	28.84 (2.37)	*p* < .001	*p* = .005	*p* < .001	*p* = .153
Reduced HDL‐C	14.56 (3.36)	28.19 (3.43)	38.78 (3.74)	26.53 (2.04)	*p* < .001	*p* = .028	*p* < .001	*p* = .076
High triglycerides	17.12 (3.43)	34.69 (4.36)	50.29 (5.37)	34.92 (2.42)	*p* = .001	*p* = .020	*p* < .001	*p* = .080
Alcohol consumption, %					*p* = .125			
Nondrinker	5.16 (1.31)	5.16 (1.17)	7.45 (1.77)	5.90 (0.69)				
Light alcohol user	80.12 (2.78)	71.68 (3.59)	63.46 (4.10)	72.14 (1.27)				
Moderate alcohol user	5.68 (2.69)	8.51 (2.69)	7.44 (2.22)	7.11 (1.08)				
Unknown	9.04 (1.86)	14.64 (2.75)	21.65 (3.57)	14.84 (1.58)				
Smoking status, %					*p* = .162			
Never	67.47 (4.39)	65.55 (4.25)	52.28 (4.60)	61.95 (2.77)				
Former	19.16 (3.28)	20.72 (3.95)	27.72 (4.63)	22.42 (1.79)				
Current	13.37 (3.09)	13.73 (2.31)	20.00 (3.21)	15.63 (1.66)				
CAP, dB/m	214.66 (3.45)	258.14 (3.84)	282.59 (4.33)	249.95 (2.55)	*p* < .001	*p* < .001	*p* < .001	*p* = .002
MASLD (yes), %	7.95 (2.24)	31.35 (2.79)	45.90 (3.72)	27.40 (2.12)	*p* < .001	*p* < .001	*p* < .001	*p* = .003

*Note*: Data is shown as weighted percentages (SE), except where otherwise noted. Characteristics of participants with different tertiles of SVR were compared by one‐way analysis of variance for continuous variables or by chi‐square test for categoric variables. Post hoc multiple comparison analysis with Bonferroni correction was performed for significant results.

Abbreviations: BMI, body mass index; CAP, controlled attenuation parameter; HDL‐C, high‐density lipoprotein cholesterol; LDL‐C, low‐density lipoprotein cholesterol; MASLD, metabolic dysfunction‐associated fatty liver disease; SVR, skeletal muscle mass to visceral fat area ratio; TC, total cholesterol; WC, waist circumference.

Participants in the highest tertile of SVR were more likely to be younger, White, and single, with more education than those in the middle tertile of SVR. Conversely, individuals in the lowest tertile of SVR tended to be older, female, more likely to be married, and less likely to be White, also having lower levels of education compared to their counterparts in the highest tertile of SVR. In comparison with participants in the middle tertiles of SVR, participants in the lowest tertiles of SVR tended to be older, female, and having lower levels of education. Moreover, the prevalence of cardiometabolic risk factors was less common among individuals in the highest and middle tertiles of SVR compared to those in the lowest tertiles of subjects. Furthermore, individuals in the highest and middle tertiles of SVR had lower BMI, WC, TC, and LDL‐C levels compared to those in the lowest tertile.

### 3.2 Associations between tertiles of SVR and MASLD

The association between tertiles of SVR and MASLD was presented in Table 2. Compared with participants in the highest tertile of SVR and after accounting for all covariates, there was significantly higher risk of MASLD for participants in middle tertile of SVR (OR: 3.11, 95% CI: 1.31–7.39, *p* = .010) and lowest tertile of SVR (OR: 3.82, 95% CI: 1.45–10.08, *p* = .007).

**TABLE 2:** Multivariable odds ratios of tertiles of SVR and MASLD.

Tertiles of SVR	Model 1		Model 2		Model 3	
	OR (95% CI)	*p*	OR (95% CI)	*p*	OR (95% CI)	*p*
Highest	Reference		Reference		Reference	
Middle	5.86 (2.98–11.51)	*p* < .001	2.42 (1.27–4.63)	*p* = .008	3.11 (1.31–7.39)	*p* = .010
Lowest	14.62 (7.81–27.39)	*p* < .001	3.47 (1.47–8.21)	*p* = .005	3.82 (1.45–10.08)	*p* = .007
*p* for trend	*p* < .001		*p* = .004		*p* = .005	

*Note*: Model 1 was adjusted for age, sex; Model 2 was adjusted for model 1 + education, race, marital status, poverty‐income ratio, BMI, WC, smoking status, and alcohol consumption. Model 3 was adjusted for model 2 + sedentary behavior, total energy intake per day, total carbohydrate intake per day, total fat intake per day, total protein intake per day, TC, and LDL‐C.

Abbreviations: BMI, body mass index; CI, confidence interval; LDL‐C, low‐density lipoprotein cholesterol; MASLD, metabolic dysfunction‐associated fatty liver disease; OR, odds ratio; SVR, skeletal muscle mass to visceral fat area ratio; TC, total cholesterol; WC, waist circumference.

**FIGURE 2:**
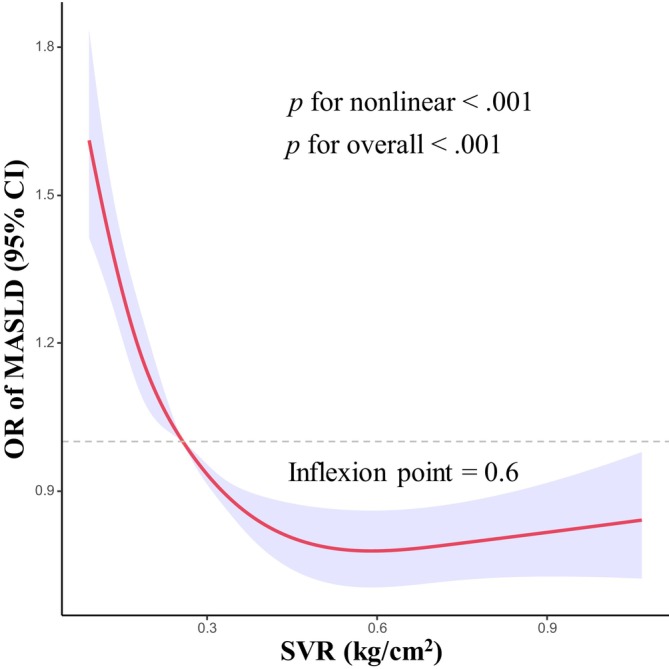
RCS plot of adjusted dose–response relationships for SVR and MASLD. The model was adjusted for age, sex, education, race, marital status, poverty‐income ratio, BMI, WC, smoking status, alcohol consumption, sedentary behavior, total energy intake per day, total carbohydrate intake per day, total fat intake per day, total protein intake per day, TC, and LDL‐C. BMI, body mass index; CI, confidence interval; LDL‐C, low‐density lipoprotein cholesterol; MASLD, metabolic dysfunction‐associated fatty liver disease; OR, odds ratio; RCS, restricted cubic spline; SVR, skeletal muscle mass to visceral fat area ratio; TC, total cholesterol; WC, waist circumference.

Nonlinear associations between the SVR and MASLD are shown in Figure 2. After adjusting for covariates, we observed an L‐shaped relationship between SVR and risk of MASLD (*p* for overall <.001). There was also evidence of nonlinearity between SVR and risk of MASLD (*p* for nonlinear <.001). The identified inflection point for the relationship between SVR and MASLD was established at 0.6 kg/cm^2^. Prior to this inflection point, the risk of MASLD decreased rapidly with increasing SVR level.

### 3.3 Sensitivity analyses

**FIGURE 3:**
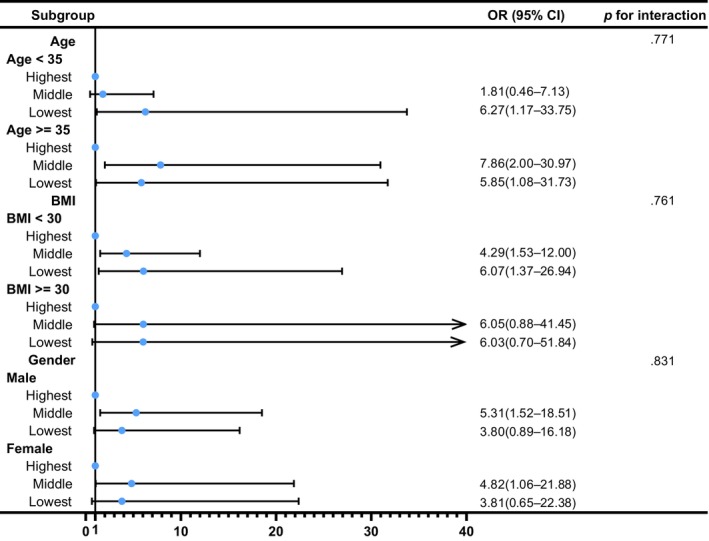
Forest plot of the relationship between tertiles of SVR and MASLD in different subgroups. Except for its stratification variables, age, sex, education, race, marital status, poverty‐income ratio, BMI, WC, smoking status, alcohol consumption, sedentary behavior, total energy intake per day, total carbohydrate intake per day, total fat intake per day, total protein intake per day, TC, and LDL‐C were adjusted. BMI, body mass index; CI, confidence interval; LDL‐C, low‐density lipoprotein cholesterol; MASLD, metabolic dysfunction‐associated fatty liver disease; OR, odds ratio; SVR, skeletal muscle mass to visceral fat area ratio; TC, total cholesterol; WC, waist circumference.

**FIGURE 4:**
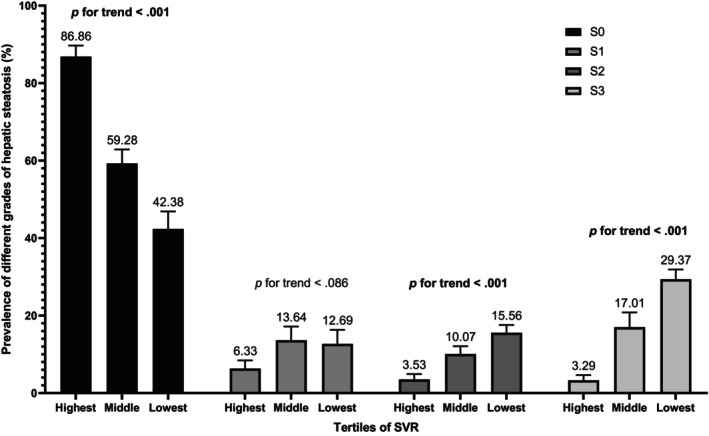
Prevalence of different grades of hepatic steatosis by SVR tertiles. SVR, skeletal muscle mass to visceral fat area ratio.

**FIGURE 5:**
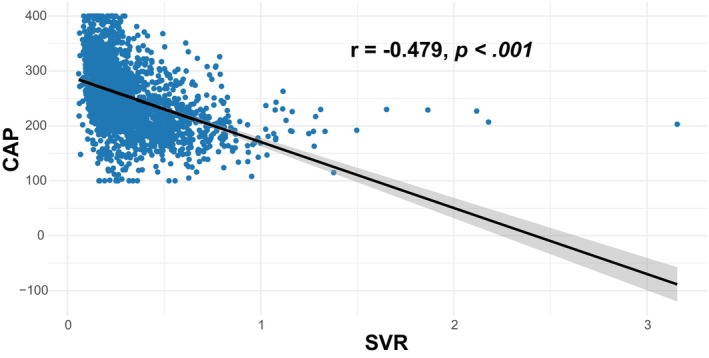
Scatter plot and Spearman correlation coefficient of CAP according to SVR. CAP, controlled attenuation parameter; r, Spearman rank correlation coefficients; SVR, skeletal muscle mass to visceral fat area ratio.

We also conducted several sensitivity analyses. In analyses using a median CAP of ≥263 dB/m for diagnosis of MASLD, the result remained robust (OR: 2.29, 95% CI: 1.14–4.64, *p* = .021 for middle tertile of SVR; OR: 2.59, 95% CI: 1.08–6.22, *p* = .034 for lowest tertile of SVR) in the Model 3 (Table S1). In addition, to determine the relationship between SVR and MASLD by age, gender, and BMI, subgroup analyses were employed. Figure 3 shows that age, gender, and BMI have no influence on the association between SVR and MASLD (all *p* for interaction >.05). Besides, Table S2 shows a similar result after multiple imputations (OR: 2.45, 95% CI: 1.25–4.81, *p* = .009 for middle tertile of SVR; OR: 3.66, 95% CI: 1.49–8.97, *p* = .005 for lowest tertile of SVR). Additionally, Figure 4 demonstrates that SVR levels are inversely associated with the prevalence of hepatic steatosis in S2 and S3 (*p* for trend <.001), but not in S1 (*p* for trend = .086). Conversely, SVR levels were positively associated with the prevalence of hepatic steatosis in S0 (*p* for trend <.001). Finally, Figure 5 shows that there is strong and significant correlation between CAP and the SVR (*r* = −0.479, *p* < .001).

## 4 DISCUSSION

The present study demonstrated that participants with higher SVR had a significantly lower prevalence of MASLD compared to those with lower SVR. Furthermore, multivariable regression analysis indicated that lower SVR significantly was associated with the higher risk of MASLD. Moreover, the relationship of SVR with MASLD was nonlinear and this finding remained robust in the sensitivity analyses.

Considering that a decrease in muscle mass may lead to an increase in visceral fat and due to the interaction between muscle mass, visceral fat, and diseases, the SVR was proposed as an index and has been proved to be useful for the estimation of risk of several diseases, which is consistent with the present study. Wang et al^22^ found that an increasing SVR was closely associated with an increased risk for exacerbating type 2 diabetes and metabolic syndrome. Lim et al^23^ conducted a survey indicating that an elevated SVR was inversely correlated with TC and LDL‐C. In our study, participants with a lower SVR tended to have a higher prevalence of cardiometabolic risk factors. These results indicated that the SVR is an excellent index for the evaluation of risk of metabolic dysregulation and cardiovascular diseases.

Besides, several studies have also examined the association between SVR and NAFLD so far. Cho et al^24^ conducted a study of South Korean adults, demonstrating that SVR was inversely associated with the risk of developing NAFLD. Unlike our study, Cho et al identified a significant interaction by sex (*p* for interaction <.001). They also found that the relative impact of the SVR on the risk of NAFLD was more pronounced in women than in men. Similarly, the research conducted by Xing et al^29^ found that the effects of SVR on the risk of some phenotype of MAFLD were more prominent in women than in men. However, the present study examined the influence of SVR on MASLD incidence and found that age, BMI, and gender did not exhibit interaction effects in this relationship, thereby reinforcing the robustness of our main analysis. The possible explanation for the difference lies in the fact that Cho et al and Xing et al used BIA to measure the body compositions, rather than DXA, which has become the reference standards to measure skeletal muscle mass and visceral fat area. BIA may result in overestimation of fat‐free mass, including muscle content, but underestimation of fat mass,^30^ thus having an impact on the estimation of SVR. Moreover, their definition of outcome was merely based on the echoes of liver ultrasound, which cannot accurately measure the liver steatosis. In another study involving 472 adults, Shida et al^31^ demonstrated that decreased muscle mass coupled with increased visceral fat mass is closely associated with an increased risk for NAFLD defined by CAP. However, Shida et al did not consider cardiometabolic risk factors, which were potential confounders and important parts for the definition for MASLD. In the present study, MASLD was defined as the presence of hepatic steatosis along with at least one of five cardiometabolic risk factors, which overcame the limitation of the study conducted by Shida et al.

SVR takes two body composition measures, skeletal muscle mass and visceral fat area, into account. Therefore, it can be used to identify SO. Although the biological mechanism between SO and MASLD is yet to be explored, there are several possible explanations. One explanation is that skeletal muscle is regarded as one of the main target organs of insulin.^32^, ^33^ Loss of skeletal muscle mass is associated with insulin resistance and diabetes, which are also linked to NAFLD.^34^ In addition, the expansion of visceral adipose tissue will lead to the change of adipokine profile.^35^ Most adiopokines, such as leptin1, resistin and retinol‐binding protein are increased, contributing to insulin resistance and inflammation.

This study had several strengths. One of them is that this study was conducted with the NHANES database, a population‐based design with a large sample size. Additionally, the present study accounted for metabolic variables, marking it as the first to evaluate the association between SVR and MASLD using updated diagnostic criteria.

However, the present study had certain limitations. First, this study was conducted with a US population; therefore, the generalizability of our findings to other populations needs to be tested. Another limitation is that we measure liver steatosis merely depending on median CAP score rather than liver biopsy, which is the gold standard method. A third limitation is that because of the limitations of the NHANES database, this was a cross‐sectional study, the causality of the relationship between the SVR and MASLD could not be determined. However, SO and MASLD share various pathophysiological mechanisms, thus establishing a cause–effect relationship between them is quite difficult. Therefore, more longitudinal studies are required to determine whether the observed relationship between the SVR and MASLD is causal.

## 5 CONCLUSIONS

The present study found an association between the SVR and MASLD in US adults. This implies that the potential effects of skeletal muscle mass and visceral fat area on MASLD should be considered during the treatment and prevention of these conditions. Moreover, our findings suggest that improving skeletal muscle mass and reducing visceral fat area may be beneficial for preventing and managing MASLD.

## AUTHOR CONTRIBUTIONS

Lisheng Wang and Hua Mao developed the study concept and design. Zhiliang Mai and Yinfei Chen analyzed and interpreted the data. Zhiliang Mai drafted the manuscript. Lisheng Wang and Zhiliang Mai revised the manuscript.

## CONFLICT OF INTEREST STATEMENT

All authors declared no potential conflict of interest relevant to the manuscript.

## Supporting information


**Table S1.** Multivariable odds ratio of tertiles of SVR and MASLD using a median CAP of ≥263 dB/m. Abbreviations: CAP, controlled attenuation parameter; MASLD, metabolic dysfunction‐associated steatotic liver disease; SVR, skeletal muscle mass to visceral fat area ratio.

**Table S2.** Multivariable odds ratio of tertiles of SVR and MASLD after multiple imputations. Abbreviations: CAP, controlled attenuation parameter; MASLD, metabolic dysfunction‐associated steatotic liver disease; SVR, skeletal muscle mass to visceral fat area ratio.

## Data Availability

All the data are available and can be freely downloaded from the National Health and Nutrition Examination Survey dataset (https://www.cdc.gov/nchs/nhanes/index.htm).
