# Supplementary material for: Association between the skeletal muscle mass to visceral fat area ratio and metabolic dysfunction‐associated fatty liver disease: A cross‐sectional study of NHANES 2017–2018
Source: J Diabetes. 2024 May 16;16(6):e13569. doi: 10.1111/1753-0407.13569 (PMC11096813; doi:10.1111/1753-0407.13569)
Supplement: Supplementary file 1 — Table S1. Multivariable odds ratio of tertiles of SVR and MASLD using a median CAP of ≥263 dB/m. Abbreviations: CAP, controlled attenuation parameter; MASLD, metabolic dysfunction‐associated steatotic liver disease; SVR, skeletal muscle mass to visceral fat area ratio. [file JDB-16-e13569-s001.docx]

**Supplementary Table S1.** Multivariable Odds Ratio of tertiles of SVR and MASLD using a median CAP of ≥ 263 dB/m.

| Tertiles of SVR | Model 1 | | Model 2 | | Model 3 | |
| --- | --- | --- | --- | --- | --- | --- |
|  | OR (95% CI) | *P* | OR (95% CI) | *P* | OR (95% CI) | *P* |
| Highest | Reference |  | Reference |  | Reference |  |
| Middle | 4.57(2.38-8.76) | *P* < 0.001 | 1.71(0.99-2.95) | *P* = 0.053 | 2.29(1.14-4.64) | *P* = 0.021 |
| Lowest | 11.30(6.54-19.54) | *P* < 0.001 | 2.41(1.34-4.34) | *P* = 0.003 | 2.59(1.08-6.22) | *P* = 0.034 |
| *P* - trend | *P* < 0.001 |  | *P* = 0.004 |  | *P* = 0.021 |  |

Model 1 was adjusted for age, sex; Model 2 was adjusted for model 1 + education, race, marital status, poverty-income ratio, BMI, WC, smoking status and alcohol consumption. Model 3 was adjusted for model 2 + sedentary behavior, total energy intake per day, total carbohydrate intake per day, total fat intake per day, total protein intake per day, TC and LDL-C.

Abbreviations: **BMI**, Body mass index; **CI**, Confidence Interval; **LDL-C**, Low-density lipoprotein cholesterol; **MASLD**, Metabolic dysfunction-associated fatty liver disease; **OR**, Odds ratio; **SVR**, skeletal muscle mass to visceral fat area ratio; **TC**, Total cholesterol; **WC**, Waist circumference.
